# Incidence and pathophysiology of gastrointestinal bleeding during mechanical circulatory support: A retrospective analysis using machine learning algorithms

**DOI:** 10.1051/ject/2025061

**Published:** 2026-03-13

**Authors:** Kelsey Gore, Dean Linder, Juan Jose Martinez Duque, Junxi Wang, Connor Rudnicki, Adrian Alexis Ruiz, Shaun Yockelson, Bobby Nossaman

**Affiliations:** 1 Department of Cardiovascular Perfusion and Extracorporeal Technology, Ochsner Health 1514 Jefferson Highway New Orleans Louisiana 70121 USA; 2 CES University, Cl 10A #22-04 El Poblado, Medellin Antioquia Columbia; 3 The University of Queensland Medical School 288 Herston Road Herston QLD 4006 Australia; 4 Critical Care Section, Anesthesiology & Perioperative Medicine, Ochsner Health 1514 Jefferson Highway New Orleans Louisiana 70121 USA

**Keywords:** Gastrointestinal bleeding, Mechanical circulatory support, Transfusion, Hemorrhage, Insulin-dependent diabetes

## Abstract

*Background*: End-organ hypoperfusion from cardiopulmonary shock may require mechanical circulatory support (MCS). However, patients receiving MCS risk the development of hemorrhagic complications, including gastrointestinal bleeding (GI). Examining potential risk factors for these complications improves clinical understanding. The purpose of this investigation was to study the risk for GI bleeding in MCS patients. *Methods*: Following IRB approval, patient characteristics, previously reported comorbidities, and the incidence of GI bleeding were reviewed from January 2017 to October 2023. Clinical variables underwent machine learning with autovalidation. Support vector machine modeling provided the best performance among the ensemble models tested. *Results*: In this study of 156 patients who underwent 284 MCS procedures, the incidence of GI bleeding was 6.0% CI 3.3–10.4%. Following machine learning, patients with insulin-dependent diabetes were associated with GI bleeding. The Receiver Operating Characteristic (ROC) curve demonstrated an area under the curve (AUC) of 0.85 with a misclassification rate of 7.5%. The relative risk of the need for major transfusion (>2 packed red blood cell units/episode) was 1.7 CI 1.1–2.5. The majority (87%), but not all, of these patients received unfractionated heparin therapy. Finally, hospital length of stay was increased in patients with GI bleeding. *Conclusion*: Insulin-dependent diabetes was associated with increased risk for GI bleeding during MCS, and these patients more often required major transfusions. Further evaluation of continuous anticoagulation therapy is warranted. Knowledge derived from this analytical study may guide the development of institutional protocols to improve care in this patient population.

## Introduction

Cardiopulmonary shock (CS) is characterized by low cardiac output and profound hypotension that can result in end-organ failure [1–4]. Patients refractory to conventional therapies, such as inotropes, vasopressors, and/or mechanical ventilation, may require mechanical circulatory support (MCS) to restore perfusion [1–5]. MCS improves deranged hemodynamics and restores perfusion to the organs. However, MCS is not without complications such as bleeding, thrombosis, and hemolysis [4–7]. The risk of bleeding is exacerbated by the administration of systemic anticoagulation medications commonly given after MCS initiation, such as unfractionated heparin [5, 6, 8].

Gastrointestinal (GI) bleeding is a recognized complication of MCS [5–9]. Its pathophysiology is multifactorial. Shear stress from MCS disrupts platelet function and impairs von Willebrand factor (vWF), predisposing to acquired von Willebrand syndrome (aVWS) [5, 10]. Low pulsatility during MCS may also cause mucosal hypoperfusion, ischemia, angiodysplasia, and eventual GI bleeding [5, 6, 9, 11]. In normal physiology, pulsatile flow regulates nitric oxide (NO) release from endothelial cells. With reduced pulsatility during MCS, impaired NO release can worsen hypotension and GI hypoperfusion [5].

This complex relationship between MCS physiology and GI bleeding warrants further investigation. Therefore, the purpose of this analytical study was to examine the preprocedural risk factors for GI bleeding in patients receiving MCS.

## Material and methods

Following IRB approval to allow electronic health records review without prior consent, data were entered into a study database. We conducted a retrospective single-center study of 156 patients who received 284 MCS devices (Appendix, Table A1) between January 2017 and October 2023 at Ochsner Health – Jefferson Highway Campus, New Orleans, Louisiana. Patients ≥18 years were included. No exclusion criteria were applied.

### Statistics

Preprocedural comorbidities and patient characteristics (Table 1) underwent machine learning with autovalidation. Model performance was assessed using AUC and misclassification rates. Support Vector Machine modeling provided the best fit. JMP Pro 18.2 was used for all statistical analyses.

**Table 1:** Baseline characteristics and previously reported co-morbidities for gastrointestinal bleeding in 156 patients requiring mechanical circulatory support.

Terms	Estimates	Std error	Chi-square	*p*-values
Intercept	–4.64	2.49	3.5	0.0625
Age	0.03	0.03	1.4	0.2297
Sex [female]	–1.12	0.69	2.7	0.1030
BMI	–0.07	0.07	1.1	0.3045
Insulin-dependent diabetes	3.51	1.54	5.2	0.0228*
Chronic renal failure	1.05	0.72	2.2	0.1405
Chronic cardiovascular disease	0.29	0.46	0.4	0.5281
Immunomodulation	0.67	0.64	1.1	0.2948
Structural lung disease	–0.51	0.44	1.3	0.2545
ICD/Pacemaker	0.23	0.52	0.2	0.6629
Atrial fibrillation	0.52	0.52	1.0	0.3169
Previous cardiac surgery	–0.14	0.43	0.1	0.7408
Congestive heart failure	–0.29	0.49	0.4	0.5537

BMI: Body mass index (kg/m^2^); ICD: Internal cardiac defibrillator; c-index statistic = 0.82; Misclassification rate = 6.0%.

## Results

**Figure 1:**
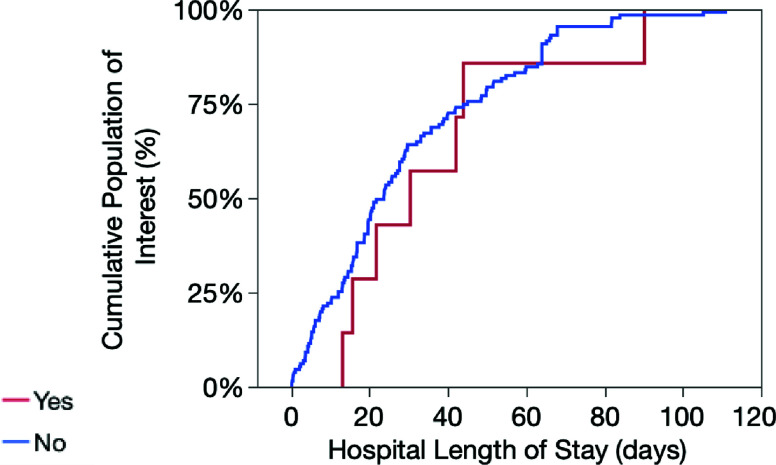
Hospital length of stay in MCS patients with GI bleeding.

**Figure A1:**
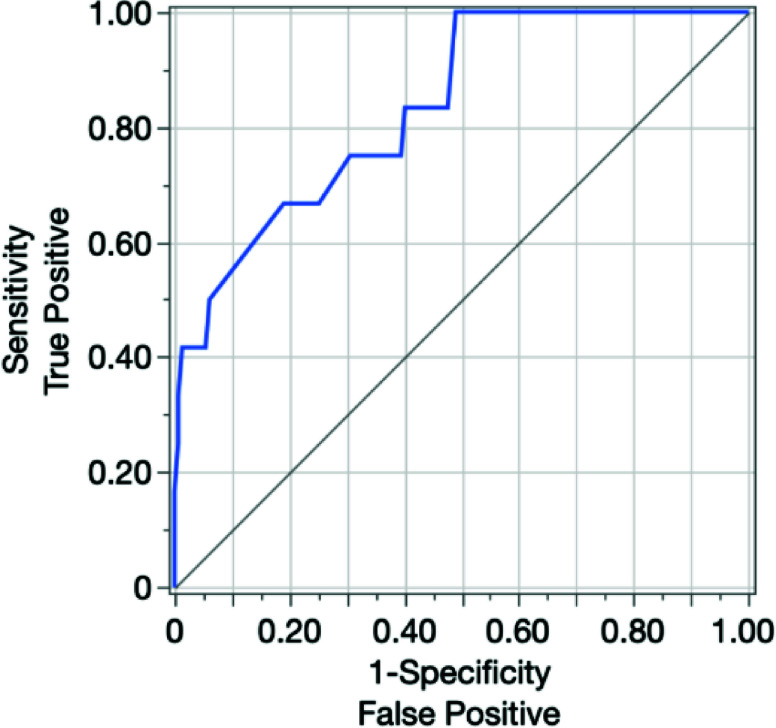
Receiver Operator Characteristic (ROC) between sensitivity and 1-specificity across classification thresholds.

The incidence of GI bleeding was 6.0% (CI 3.3–10.4%). Insulin-dependent diabetes was associated with increased risk of GI bleeding (Table 1). Our institution applies insulin-guided protocols for glycemic management before and during MCS. The ROC curve for the Support Vector Machine model (Appendix, Figure A1) demonstrated an AUC of 0.85 and a misclassification rate of 7.5%. The curve shows sensitivity versus 1-specificity. The steep initial rise indicates strong discriminative power, and the AUC demonstrates performance substantially above random (grey diagonal). The step-like pattern reflects limited data, highlighting the need for validation in larger cohorts. Patients with GI bleeding were more likely to require major transfusion (>2 units pRBC/episode), with a relative risk of 1.7 (CI 1.1–2.5). Most (87%) received unfractionated heparin therapy in accordance with institutional guidelines. GI bleeding was also associated with prolonged hospital length of stay (Figure 1).

## Discussion

This investigation is, to our knowledge, the first report of increased GI bleeding among insulin-dependent diabetic patients receiving MCS across a range of device types. MCS confers substantial bleeding risk [6], and comorbidity profiles should guide individualized therapies. Further investigation of anticoagulation protocols, particularly continuous therapy, is warranted. GI bleeding was defined as hematochezia, hematemesis, melena, bloody NG output, transfusion requirement, or active bleeding observed at endoscopy. Patients with GI bleeding had nearly twice the relative risk of requiring major transfusion [>2 units of packed red blood cells (pRBC)]. Transfusion may control bleeding but can worsen systemic inflammation, particularly in diabetic patients with baseline mucosal vulnerability [12, 13]. Prior studies, including Kapuria et al., have demonstrated higher rebleeding rates in diabetic patients on MCS [10].

In our cohort, GI bleeding was also associated with longer hospitalization, consistent with prior studies [6]. Together, these findings support the importance of comorbidity assessment in risk stratification for MCS patients.

## Limitations

This study has limitations inherent to a retrospective single-center design. Data completeness, though strengthened by electronic medical records, may be imperfect. Another limitation is the lack of device-type analysis. Our current sample size does not permit robust statistical comparisons between device types. However, all MCS devices share intravascular access to the systemic circulation, triggering inflammation and activation of the coagulation cascade – a mechanism particularly relevant in insulin-dependent diabetes. Future studies should prioritize device-specific analyses.

## Conclusions

Insulin-dependent diabetes was associated with an increased risk for GI bleeding during MCS. Patients with this complication more often required major transfusions and experienced longer hospital stays. This suggests the need for further investigation into anticoagulation strategies. Knowledge derived from this analytical study may inform institutional protocols to improve outcomes in this population.

## Data Availability

All available data are incorporated into the article.

## Appendix

**Table A1:** Acute and durable mechanical circulatory devices within our study cohort.

Acute MCS Devices
IABP*
Impella
TandemHeart
ECMO*
BiVAD*
RVAD*
Durable MCS Device
LVAD*
Total

*Intra-aortic balloon pump; Extracorporeal Membrane Oxygenation; Biventricular assist device; Right ventricular device; Left ventricular device, respectively.
